# Early Radiological Outcome of Hallux Valgus Corrective Surgery With an Intramedullary Plate: A Single-Center Case Series

**DOI:** 10.7759/cureus.67965

**Published:** 2024-08-27

**Authors:** Soo May Lim, Ewe Juan Yeap

**Affiliations:** 1 Department of Orthopaedics and Traumatology, Hospital Raja Permaisuri Bainun, Ipoh, MYS; 2 Department of Orthopaedics and Traumatology, ParkCity Medical Centre, Kuala Lumpur, MYS

**Keywords:** bosch osteotomy, intramedullary plate, radiological outcome, mini-open, hallux valgus surgery

## Abstract

Introduction: Hallux valgus is a common foot deformity that can lead to significant pain and functional limitations. Minimally invasive corrective surgery is becoming increasingly popular. The aim of this study is to evaluate the radiological outcome of mini-open hallux valgus surgery using an intramedullary plate in Asian patients.

Methods: A series of seven patients (six females, one male) and 10 feet underwent hallux valgus correction surgery. Three of the patients had bilateral surgery. Age ranged from 31 to 54 years old. All patients had severe pain and functional limitations. The cases are mild to moderate in severity, of which the radiological parameters are the hallux valgus angle (HVA), intermetatarsal angle (IMA), and distal metatarsal articular angle (DMAA). A Bosch osteotomy (distal metatarsal osteotomy) is performed, and a locking plate was inserted into the medullary canal with the distal part of the plate displacing the metatarsal head laterally. The rotational deformity was corrected. The plate is fixed to the metatarsal head. Akin's osteotomy was performed in all cases. Patients were allowed to bear weight immediately after surgery and were followed up at regular intervals with serial radiographs.

Result: The follow-up period was three months. All patients were full weightbearing with minimal or no pain at three months. Wounds were well healed. Osteotomy sites were united, with significant radiological improvement (mean HVA: 24.1° to 7.2°; mean IMA: 17° to 7.8°; mean DMAA: 13.7° to 4.2°).

Conclusion: Mini-open hallux valgus surgery using an intramedullary plate is a safe and effective technique that can lead to significant improvement in pain and function for mild to moderate hallux valgus. The modified placement of the plate into the medullary canal allows for a smaller incision while providing correction of the hallux valgus deformity.

## Introduction

In recent years, more than 200 surgical techniques have been described for the correction of hallux valgus deformity [1,2]. The primary objective of hallux valgus surgery is to reduce the deformity and restore a pain-free and functional forefoot. Various surgical techniques are available for this purpose, including soft tissue procedures, first metatarsal osteotomies at different levels, osteotomies in the first cuneiform, arthrodesis, etc. [3,4]. Proximal osteotomies of the first metatarsal effectively correct the intermetatarsal angle (IMA) but do not address the distal metatarsal articular angle (DMAA). Diaphyseal osteotomies allow the correction of both angles. Distal metatarsal osteotomies are suitable for mild to moderate hallux valgus as they offer limited correction of the IMA compared to proximal osteotomies [5].

One of the popular techniques that have been extensively utilized is distal subcapital osteotomy of the first metatarsal bones and lateral displacement using an axial Kirschner wire (K-wire) for fixation, described by Bösch, as an adaptation of Kramer's technique [6]. However, the usage of K-wire is associated with multiple complications such as pin tract infection, first metatarsophalangeal joint (MTPJ) stiffness, loss of reduction, etc. Surgeons then developed techniques to address these limitations. Screws were subsequently favoured over K-wires to hold the correction. Several authors have also described the use of intramedullary devices and plates to stabilize the osteotomy [7-10].

This article describes a modification of Bösch's technique that involves replacing the axial K-wire by a small fragment locking compression plate (LCP) (Osteomed FPS Foot Plating System, Addison, TX) as an intramedullary device to stabilize transverse osteotomies of the distal first metatarsal for the correction of mild to moderate hallux valgus deformity. This allows a more stable fixation of the osteotomy with greater rotational and dorsoplantar stability [10] and is performed via a mini-open approach. A limited number of papers describe the use of intramedullary devices in Asian patients [11-13], and this is the first case series on the use of small fragment LCP in hallux valgus surgery in this population. The aim of this article is to present primarily the initial results of this technique in terms of improvement of radiological parameters, which are the hallux valgus angle (HVA), IMA, and DMAA, and secondarily clinical outcomes and complications and to compare it with the results of other techniques.

## Materials and methods

This is a retrospective study conducted at a single center. Patients who had undergone hallux valgus corrective surgery using an intramedullary plate between November 2022 and September 2023 were identified and included in the study. Inclusion criteria were adult patients with symptomatic hallux valgus who did not respond to a trial of conservative management and had undergone hallux valgus corrective surgery with an intramedullary plate. In contrast, patients who have arthritis of the big toe MTPJ and deformities of the hindfoot or midfoot or other toes, those who were revision cases, or those who had undergone surgery with alternative methods of stabilization of osteotomy were excluded. All procedures were performed by a single person (senior author). 

Surgical procedure

A 2.5 cm skin incision is made at the medial aspect of the first MTPJ in the transition of dorsal and plantar skin, over the medial utility approach. Arthrotomy is done in an elliptical manner, and the medial prominence of the metatarsal head is then exposed. Bunionectomy is performed with a microsagittal saw, with minimal bone resected to maximize laterization. Lateral capsulotomy is done in a blind method through the same incision. Hohmann retractors are placed dorsal and plantar proximal to the first MTPJ. Under direct vision, a transverse osteotomy is performed with a microsagittal saw at the neck of the first metatarsal, extra-articular and perpendicular to the axis of the shaft in the sagittal plane. The metatarsal head was then manipulated (surgeon holding onto the big toe), translated laterally to correct the HVA and IMA, as well as rotated in the coronal and axial plane, correcting the DMAA and pronation deformity, respectively. 

Once the desired correction was obtained, a 2.4 mm or 2.7 mm subcondylar mini-locking plate (Osteomed Foot Plating System) was inserted into the intramedullary canal. Locking screws were then inserted and locked into the metatarsal head, with a cortical screw inserted into the proximal shaft. Satisfactory correction of HVA, IMA, DMAA, and implant placement was confirmed under the image intensifier. An Akin's procedure or medial closing wedge osteotomy of the proximal phalanx of the big toe was performed if there was hallux valgus interphalangeus. The medial capsule with the elliptical opening was then closed with polydioxanone suture size USP-1 (United States Pharmacopeia size 1, North Bethesda, MD) absorbable sutures to reinforce the stability of the osteotomy site and correct the sesamoid position if needed. Soft tissue and skin were closed in layers. 

**Figure 1:**
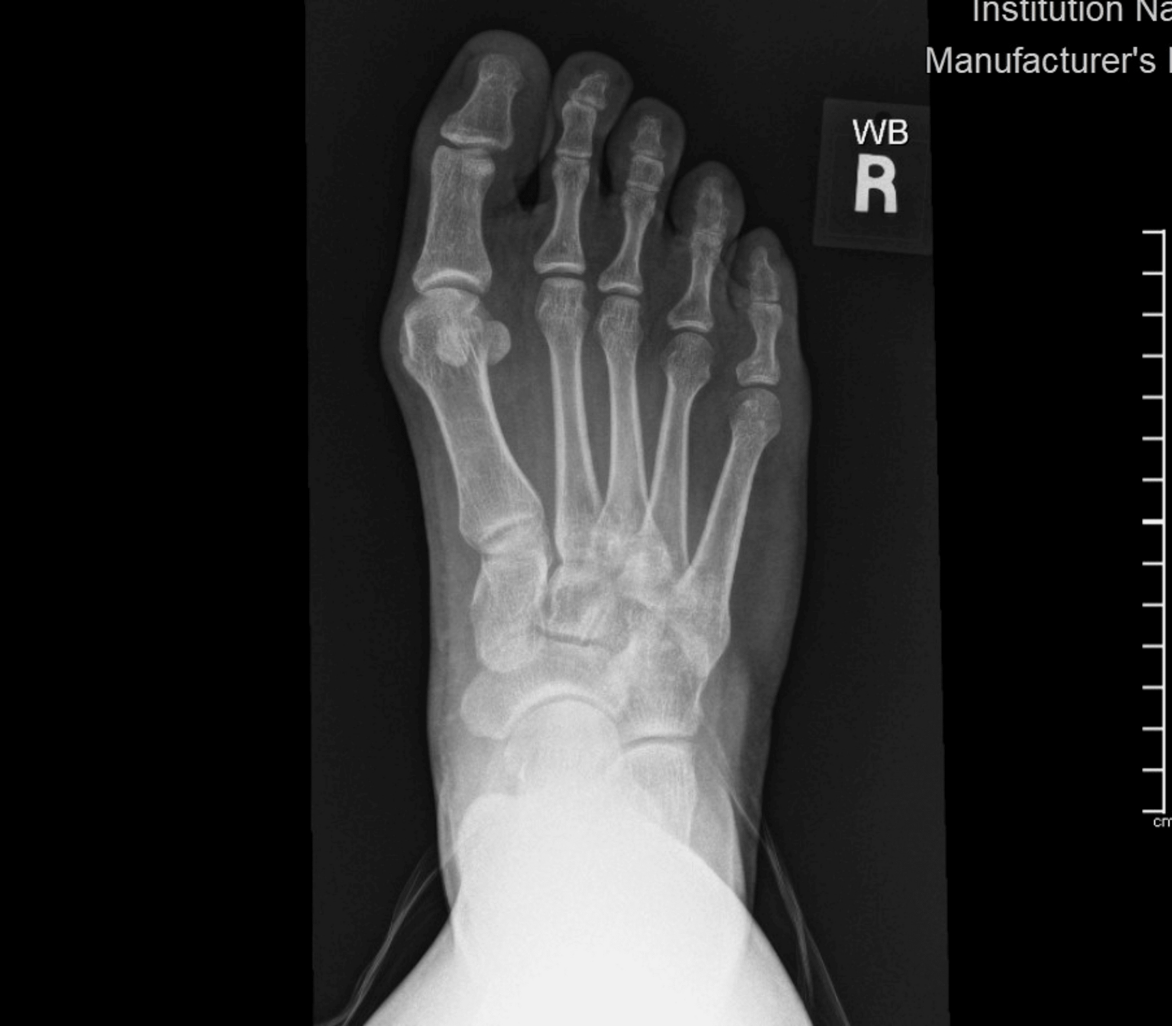
Pre-operative weightbearing AP radiograph AP: anterior-posterior

**Figure 2:**
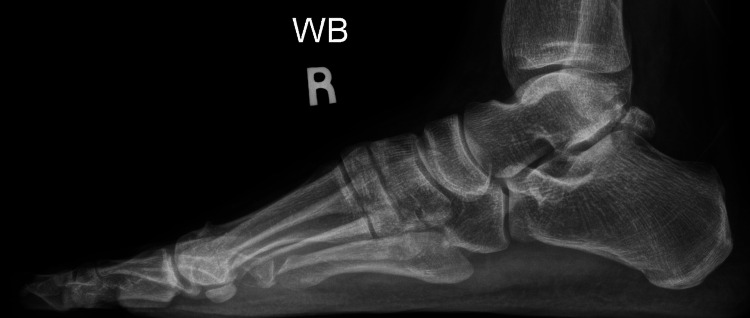
Pre-operative weightbearing lateral foot radiograph

**Figure 3:**
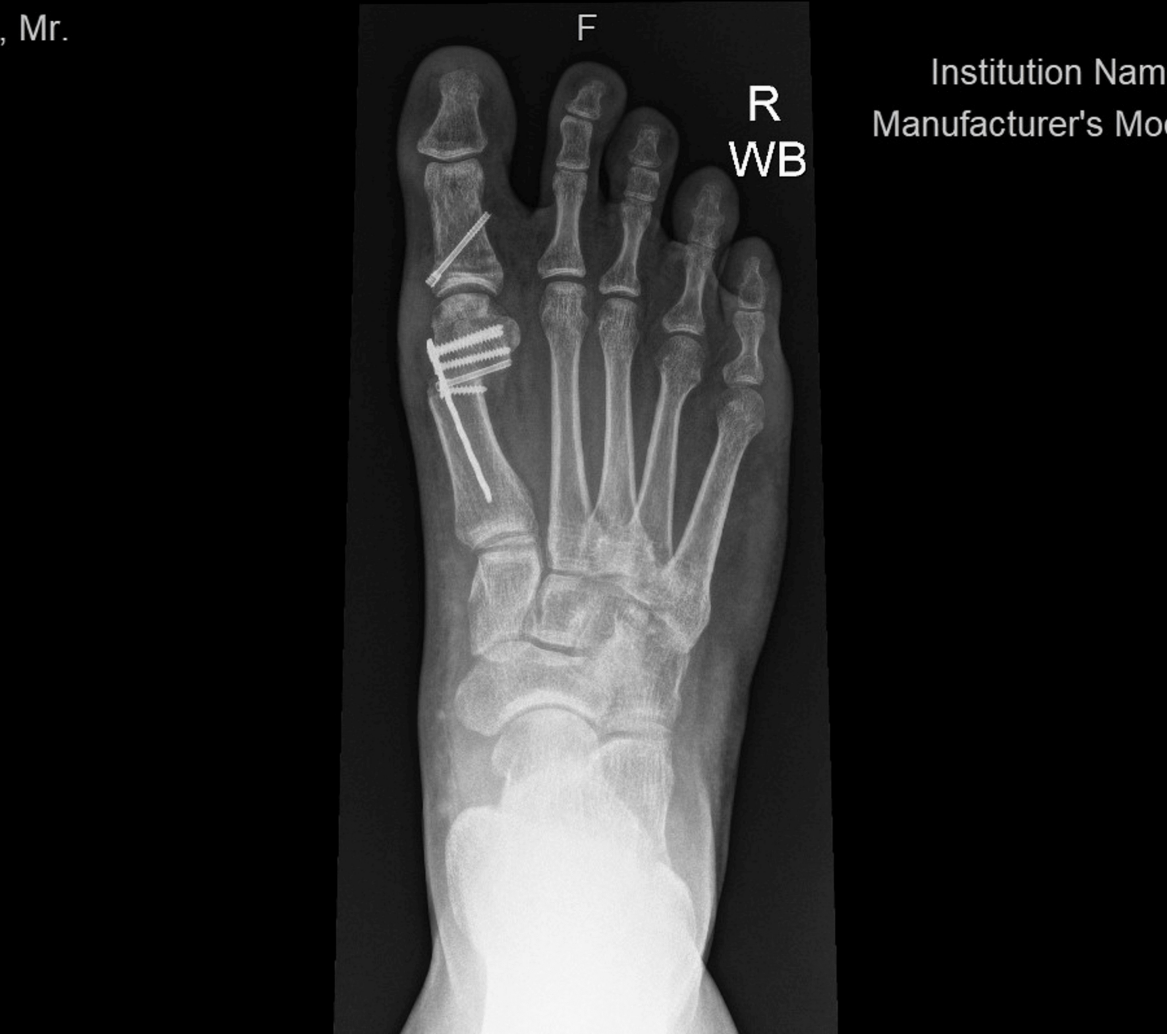
Post-operative weightbearing AP foot radiograph showing the intramedullary plate inserted AP: anterior-posterior

**Figure 4:**
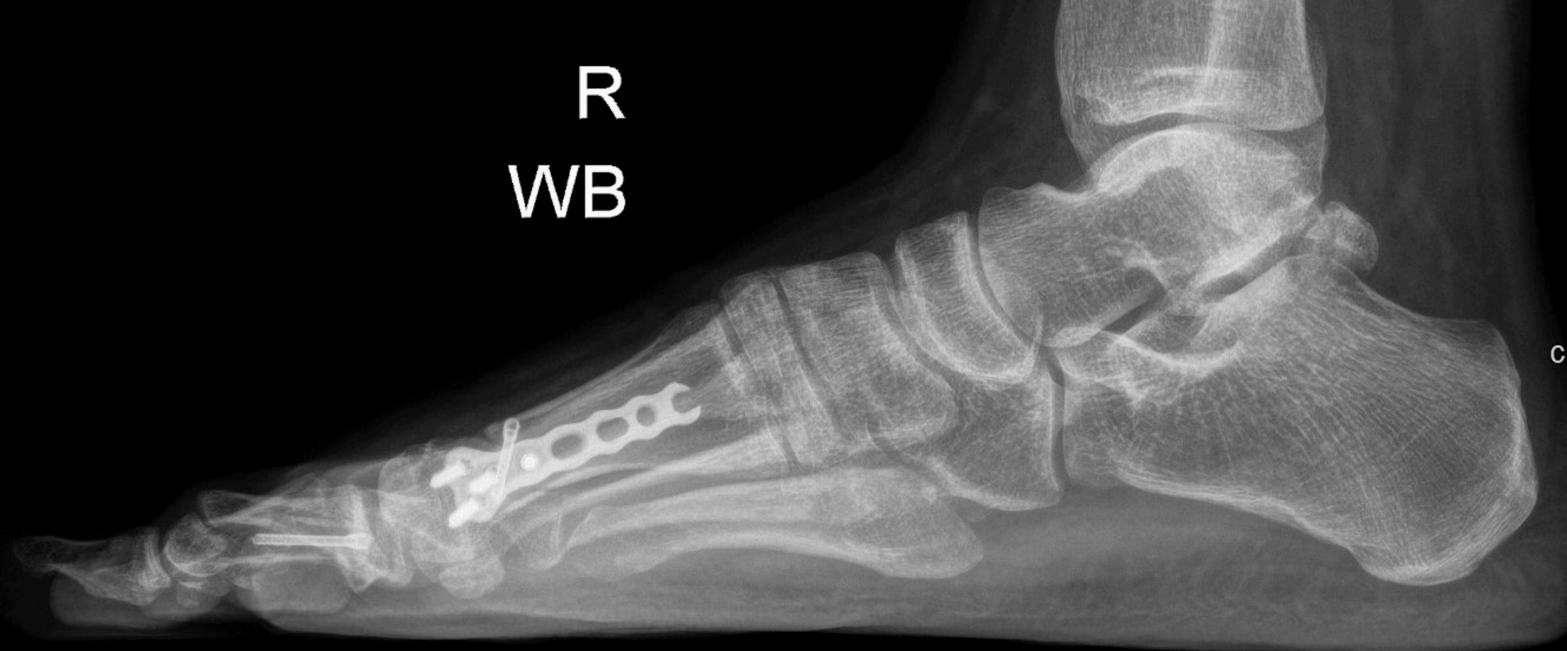
Post-operative weightbearing lateral foot radiograph showing the intramedullary plate inserted

Weightbearing anterior-posterior (AP) or dorsoplantar and lateral view foot radiographs were obtained pre-operatively, immediately post-op, and then at monthly follow-ups until three months. Figure 1 and Figure 2 show the pre-operative weightbearing AP and lateral radiographs of a patient, while Figure 3 and Figure 4 show the three-month post-operative AP and lateral radiographs of the same patient. On the AP radiographs of the foot, the pre-operative and post-operative HVA, IMA, and DMAA were measured. The intersection of the longitudinal axes of the first metatarsal bone and the big toe proximal phalanx is the HVA. The IMA was defined as the angle subtended between the longitudinal axes of the first and second metatarsal bones [14]. The longitudinal axis of the proximal phalanx refers to a line connecting two points, which are the proximal and distal midpoints in the proximal phalanx. The distal midpoint is taken at 0.5 cm proximal to the most distal articular aspect on the head, whereas the proximal midpoint is 0.5 cm distal to the articular surface of the base. Likewise, the longitudinal axis of the metatarsal refers to the line connecting the two proximal and distal midpoints. The distal midpoint is taken at 1 cm proximal to the most distal articular aspect on the head, whereas the proximal midpoint is 1 cm distal to the articular surface of the base [11]. The DMAA defines the relationship of the articular surface of the distal first metatarsal in relation to the first metatarsal bone longitudinal axis and indicates if the MTPJ is congruent. The DMAA is the angle subtended between the first metatarsal longitudinal axis and a line perpendicular to the lateral slope of the metatarsal head (medial and lateral limits of the articular surface) [14]. 

**Figure 5:**
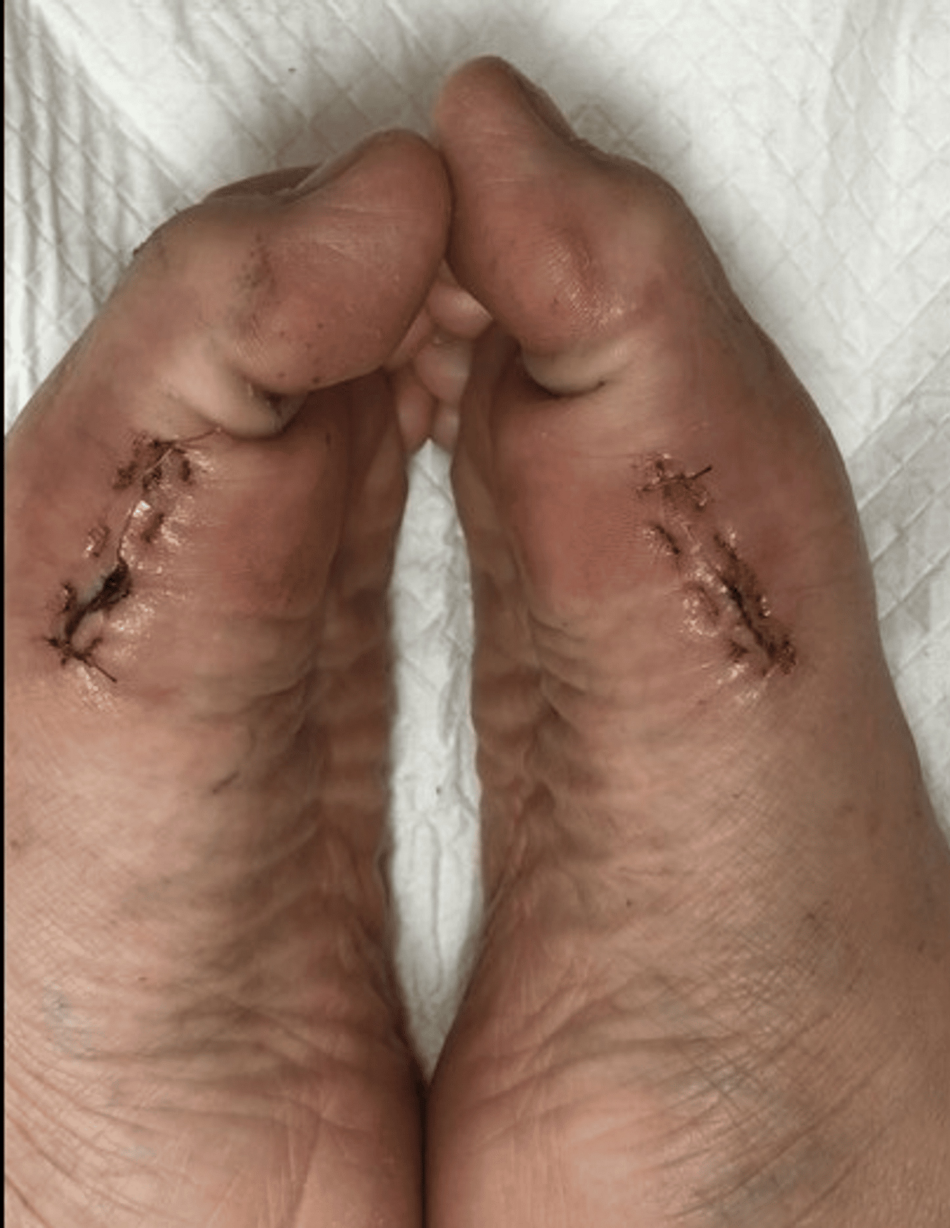
Active bilateral MTPJ flexion two weeks post-operatively MTPJ: metatarsophalangeal joint

**Figure 6:**
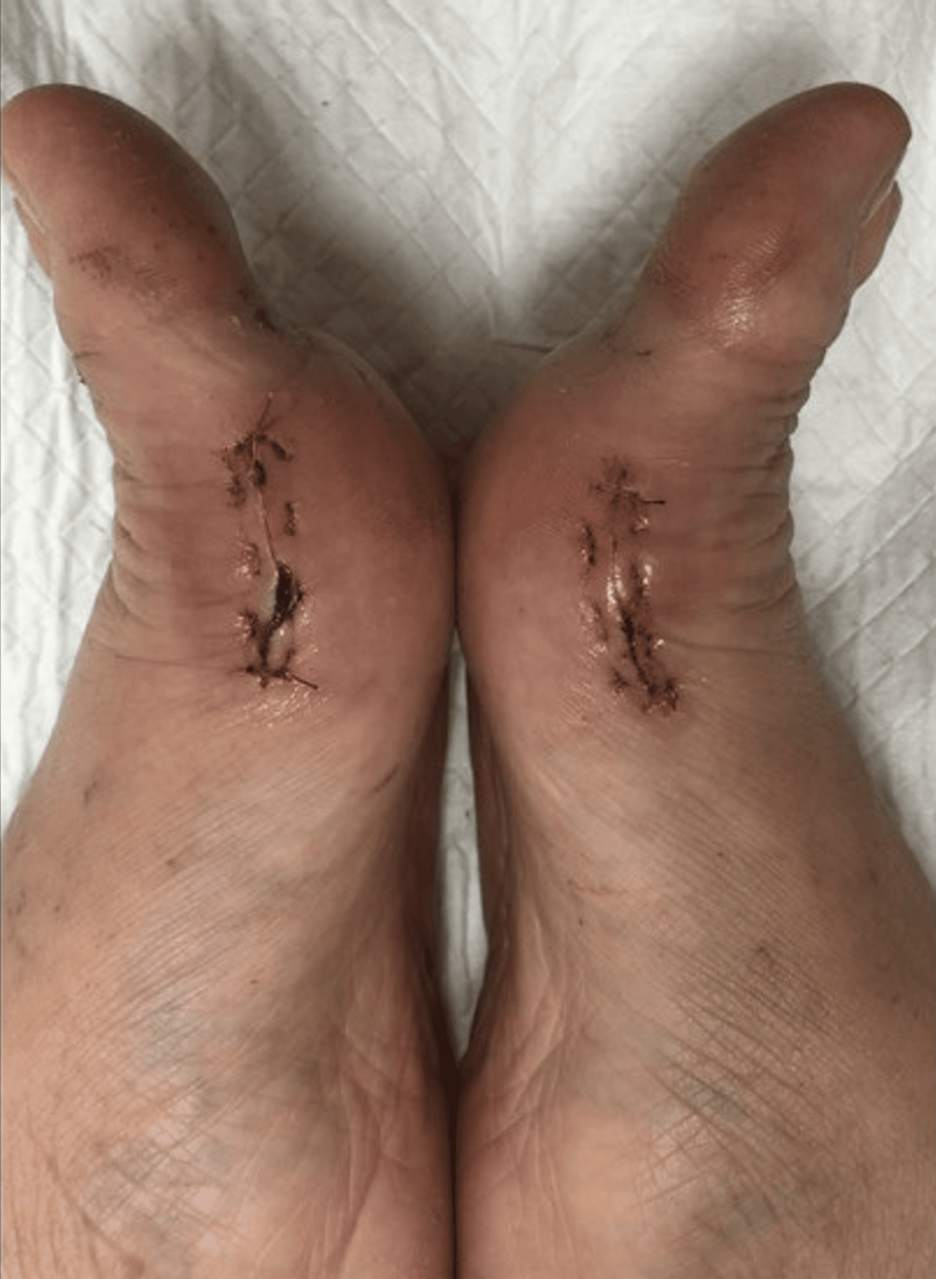
Active bilateral MTPJ extension two weeks post-operatively MTPJ: metatarsophalangeal joint

Post-operatively, dressing is done with extra padding between the big toes and second toe to prevent the recurrence of the deformity. This is maintained until two weeks later. Range of motion exercises of the first metatarsal phalangeal joint were started immediately post-op as tolerated by the patient. Figure 5 and Figure 6 demonstrate the range of motion two weeks post-op. Full weightbearing is allowed wearing a DARCO OrthoWedge shoe after the first post-operative day, and the shoe was kept for four weeks post-operatively. Henceforth, normal shoe wear is allowed.

The pre-operative and post-operative three-month HVA, IMA, and DMAA measurements for all patients were analyzed and compared, and a mean pre- and post-value was derived for each. Data normality was tested with the Shapiro-Wilk test, and a p-value above 0.05 was set to show a normal data distribution. The paired t-test was used for comparison of the pre- and post-operative data, and a p-value below 0.05 was set to show a statistically significant comparison. The statistical analysis was performed with IBM SPSS Statistics for Windows, V. 26.0 (IBM Corp., Armonk, NY).

## Results

A total of seven patients (10 feet) were identified, six of whom were female and one was male. Three patients had undergone bilateral surgery. Their age ranged from 31 to 54 years old. Demographic data of the subjects are displayed in Table 1.

**Table 1:** Demographic data of patients

Characteristics	Value
Total patients (persons)	7
Male patients (person)	1
Female patients (persons)	6
Total number of feet operated on (feet)	10
Left side (feet)	5
Right side (feet)	5
Average age (years)	49.9

The pre- and post-operative radiological measurements are presented in Table 2. The HVA improved from a mean of 24.1° pre-operatively to a mean of 7.2° post-operatively (p<0.05). The IMA improved from a mean of 15.8° to 7.8° (p<0.05), while DMAA improved from 13.7° to 4.2° (p<0.05). There were no cases of osteotomy nonunion or avascular necrosis of the metatarsal head at the three-month follow-up. No wound complications or infection was seen in any patient.

**Table 2:** Radiographic changes pre- and post-operatively HVA: hallux valgus angle; IMA: intermetatarsal angle; DMAA: distal metatarsal articular angle

Parameter	Pre-op mean (degrees)	Pre-op range (degrees)	Post-op mean (degrees)	Post-op range (degrees)	Mean correction (degrees)
HVA	24.1	16.2-31.6	7.2	1.4-12.6	16.9
IMA	15.8	11.4-20.6	7.8	4.8-9.5	8.0
DMAA	13.7	4.2-20	4.2	1.1-9.2	9.5

## Discussion

In the past decade, there has been a development of various types of intramedullary devices for the fixation of distal metatarsal osteotomy fragments, and several authors have shown good radiological and functional results with such devices [7,8,15-17]. One of the first few authors to describe this technique, Palmanovich and Myerson, described that using the intramedullary locking plate known as Mini MaxLock Extreme ISO (Intraosseous Sliding Osteotomy), a high lateral translation and stable fixation of the metatarsal head could be obtained even in some of the severe hallux valgus deformities [7]. Subsequently, in 2015, with a prospective study of 57 patients, Bennett and Sabetta showed the ISO to be a low-profile but strong and stable implant that was easy and had good radiological and American Orthopaedic Foot and Ankle Society (AOFAS) functional outcomes at one-year follow-up [15]. In 2015, Biz et al. had shown excellent results with another intramedullary nail device known as the Endolog in 30 patients who were followed up for four years [17]. Their results were consolidated by a follow-up study of a larger sample size (100 patients) in 2021 [9]. Yet another popular intramedullary device that had shown similar success in treating hallux valgus with minimal complications is the V-TEK intramedullary locking plate that offers a few different offsets to maximize lateral translation [10,18,19].

An intramedullary device has certain advantages over other methods, in terms of union, stability of fixation, recovery time, and weightbearing [7]. Its corrective abilities are highly versatile as the metatarsal head is directly visualized, allowing the correction of deformity in all planes. In the dorsal-plantar plane, plantarization of the metatarsal head can be controlled, and pronation deformity can be corrected in the rotational axis. In the medial-lateral plane, HVA and IMA can be reduced, with or without adjunct procedures like lateral soft tissue release and Akin's procedure. In addition, in the axial plane, DMAA abnormalities can also be addressed [9]. The correction of deformity is maintained by a biomechanically stable device which provides strong fixation at the osteotomy site, leading to faster bone healing and enabling early weightbearing. All the intramedullary devices permitted immediate weightbearing with forefoot offloading shoes. It also avoids the use of cast or K-wires that transfixes the big toe MTPJ that may cause joint stiffness and pin site infection. Early range of motion exercises of the big toe MTPJ are possible. These factors contribute to faster healing and a quicker return to normal activities of daily living.

The use of a mini-LCP for hallux valgus corrective surgery has limitations, such as a limited lateral translation of the metatarsal head due to its flat design and lack of an offset, rendering it only suitable for mild to moderate cases [20]. However, the additional benefits offered by this technique include the cheap cost (compared to other intramedullary devices), easy availability for application and hence removal (does not require special sets or instrumentations which are not available in some countries), and faster learning curve as this is an implant familiar to most surgeons. In addition, as there are multiple locking holes, it may be more suitable for cases of osteoporotic bones or conditions where there are cystic changes within the metatarsal head, for example, rheumatoid arthritis. There is also potential for it to be used as a salvage implant if there are complications intra- or post-operatively following other hallux valgus correction surgeries like percutaneous screw fixation, such as screw cut-out or re-displacement of correction, especially in cases with inadequate bone stock. Furthermore, as demonstrated in a recent study by Uluoz and Gökmen in 2024, the extent of metatarsal head lateralization and degree of DMAA correction can be controlled by the angle of insertion and progressive tightening of the cortical screw which is fixed to the proximal shaft of the first metatarsal [21]. It may also avoid complications associated with other techniques such as that associated with percutaneous screw fixation, including articular surface disruption, failure to obtain the stable "in-out-in" fixation in cases of metatarsus adductus or narrow foot, etc. [11,21,22].

In recent years, minimally invasive surgery (MIS) or percutaneous approaches have gained popularity due to the advantages of a smaller skin incision and hence better scar cosmesis, less post-operative pain, earlier mobilization, shorter hospital stays and quicker recovery, less soft tissue trauma, and hence less wound infection or breakdown. It would seem beneficial especially for patients with wound healing concerns, such as patients with skin conditions, diabetes, or peripheral vascular disease. 

Minimally invasive hallux valgus surgery is constantly evolving and has currently advanced to the third generation with several clinical studies demonstrating excellent outcomes through minimally invasive or percutaneous osteotomy and stable internal fixation. Some studies like that of Kaufman et al. have shown similar clinical and radiological outcomes between minimally invasive and open hallux valgus surgery in a study of five-year follow-up [23]. However, a meta-analysis of 22 studies by Ji et al. in 2022 had shown less pain at the early phase, a shorter surgery time, and a shorter hospitalization time in the MIS group compared with the open group [24]. 

A further differentiation had been made between the terms percutaneous surgery and MIS. Roukis had defined percutaneous surgery as incisions shorter than 5 mm, requiring specific surgical tools like mini blades for soft tissue and Shannon burr, whereas MIS incisions are within 2 cm and use traditional equipment like the microsagittal saw [25]. Fluoroscopy is essential in the former group, while it may be omitted in the latter group [24]. Di Giorgio et al. had defined MIS as that with an incision of 3 cm in both his studies on the Endolog system [8,26]. Our study utilized an incision of 2.5 cm for osteotomy and insertion of an implant. However, there is a possibility that the size of this incision can be reduced over time in the future, as demonstrated by Tang et al. who had utilized a 2 cm incision for the intramedullary Spear plate system [11]; by Fernandez who utilized an incision of 5 mm for hallux valgus surgery using the V-TEK intramedullary locking plate system [10]; and in a case report by Cho et al. who utilized an 8 mm incision using a low-profile LCP [12].

The outcomes of our surgical technique have been shown to be similar to recent studies that analyzed the radiological outcome of newer minimally invasive surgical techniques with comparable pre-operative radiological parameters as our patients. A study by Rodriguez-Materon et al. in 2023 of 95 patients who underwent fourth-generation MIS showed a post-operative HVA of 8.4° and IMA of 6.9° [27]. Another study in 2024 by Kim et al. yielded a post-operative HVA of 7.3°, IMA of 13.1°, and DMAA of 22.3° [28], for patients who underwent percutaneous hallux valgus surgery.

The limitation of our study is the lack of functional outcome to determine the clinical efficacy of the procedure, small sample size, and short follow-up duration. A longer follow-up duration would be necessary to detect other potential problems such as avascular necrosis of the metatarsal head, stiffness of the MTPJ, and soft tissue irritation of the implant that may necessitate removal. As this paper describes the early outcome of a new technique, it serves as a pilot project for a prospective study with a larger sample size and longer follow-up duration that will assess the radiological and functional outcomes.

## Conclusions

The easily available and low-cost mini fracture locking plate placed intramedullary for a distal metatarsal osteotomy may be a safe and effective option for mild to moderate hallux valgus surgery in certain indications. It allows for the accurate correction of deformity and stable fixation of osteotomy, using a mini-open approach that minimizes soft tissue disruption.
